# Supplementary material for: Population Age Structure and Greenhouse Gas Emissions from Road Transportation: A Panel Cointegration Analysis of 21 OECD Countries
Source: Int J Environ Res Public Health. 2020 Oct 22;17(21):7734. doi: 10.3390/ijerph17217734 (PMC7672923; doi:10.3390/ijerph17217734)
Supplement: Supplementary file 1 [file ijerph-17-07734-s001.pdf]

Supplementary Materials

Table A1. Country list.

Code	Name	Code	Name
**AUT**	Austria	HUN	Hungary
**BEL**	Belgium	ITA	Italy
**CAN**	Canada	JPN	Japan
**CHE**	Switzerland	KOR	Korea, Rep.
**DEU**	Germany	NLD	Netherlands
**DNK**	Denmark	NOR	Norway
**ESP**	Spain	POL	Poland
**FIN**	Finland	PRT	Portugal
**FRA**	France	SWE	Sweden
**GBR**	United Kingdom	USA	United States
**GRC**	Greece		

Table A2. Unit root test results.

Variable	Im-Pesaran-Shin (IPS)	Augmented-DF (ADF)	Phillips-Perron (PP)	Cross-sectionally augmented-IPS (CIPS)	Results
E **(emissions)**	0.517	0.943	3.259	−1.193	I (1)
Y **(income)**	−0.455	−0.329	−0.303	−2.077 *	I (1)
FP **(fuel price)**	−0.227	−0.183	−0.222	−1.735	I (1)
VO **(vehicle ownership)**	−0.917	−0.884	−0.215	−1.996	I (1)
PD **(population density)**	4.348	4.724	7.045	−1.405	I (1)
UR **(urbanization)**	1.863	2.238	−1.023	−1.058	I (1)
FE **(fuel economy)**	4.043	4.393	4.709	−1.172	I (1)
TV **(transport volume)**	−1.322 *	0.113	1.772	−2.150 *	I (1)
FT **(fuel transition)**	6.524	6.117	6.557	−1.455	I (1)

Note: * p-value < 0.10.

Table A3. Cointegration test results.

Test	Cointegration test		
	**Modified Dickey-Fuller (DF) test**	**DF**	**Augmented DF**
**Group 1 (z1 and *E*, *Y*, *FP*, *VO*, *PD*, *UR*, *FE*, *TV*, *FT*)**	−1.928 **	−3.008 ***	−4.827 ***
**Group 2 (z2 and *E*, *Y*, *FP*, *VO*, *PD*, *UR*, *FE*, *TV*, *FT*)**	−2.062 **	−3.067 ***	−4.870 ***
**Group 3 (z3 and *E*, *Y*, *FP*, *VO*, *PD*, *UR*, *FE*, *TV*, *FT*)**	−2.574 ***	−3.306 ***	−5.089 ***

Note 1: this test uses the demeaned estimator of a Kao cointegration test**.** Note 2: groups 1–3 have *E*, *Y*, *FP*, *VO*, *PD*, *UR*, *FE*, *TV*, *FT* in common, and each group includes *z1*, *z2*, *z3*, respectively. Note 3: *** p-value < 0.01, ** p-value < 0.05
